# Cytogenomic Abnormalities in Children With Acute Lymphoblastic Leukemia From Western Mexico: A Single‐Center Fluorescence In Situ Hybridization‐Based Study

**DOI:** 10.1002/jha2.70220

**Published:** 2026-01-19

**Authors:** Rosa María González Arreola, María Teresa Magaña Torres, Ma. Guadalupe Domínguez Quezada, Janet Margarita Soto Padilla, José Luis Toro Castro, Beatriz Kazuko De la Herrán Arita, Alicia Gutiérrez Méndez, Hugo Antonio Romo Rubio, Juan Ramón González García

**Affiliations:** ^1^ Doctorado en Genética Humana Centro Universitario en Ciencias de la Salud Universidad de Guadalajara Guadalajara Jalisco Mexico; ^2^ División de Genética Centro de Investigación Biomédica de Occidente Instituto Mexicano del Seguro Social Guadalajara Jalisco Mexico; ^3^ Servicio de Hematología UMAE‐Hospital de Pediatría Centro Médico Nacional de Occidente Instituto Mexicano del Seguro Social Guadalajara Jalisco Mexico

**Keywords:** cytogenomic abnormalities, fluorescence in situ hybridization, mexican population, overall survival, pediatric ALL

## Abstract

**Introduction:**

In Mexico, the 5‐year overall survival (OS) rate for pediatric acute lymphoblastic leukemia (ALL) ranges from 45% to 85%, markedly lower than the ∼90% reported in high‐income countries, where cytogenomic testing is essential for accurate risk stratification and therapeutic decision‐making. The few available data for Mexican cohorts derive from studies conducted in Mexico City using conventional karyotyping, DNA index analysis, and RT‐PCR targeting only four gene fusions. Broader cytogenomic characterization is needed to identify additional prognostic alterations.

**Methods:**

We analyzed 170 pediatric ALL cases (150 B‐Cell lineage, 10 T‐Cell lineage, and 10 mixed phenotype) using fluorescence in situ hybridization (FISH) with a panel of 11 probe sets targeting recurrent cytogenomic abnormalities. All patients were treated according to the Total XV protocol.

**Results:**

Among 150 B‐Cell ALL cases, recurrent cytogenomic abnormalities included ETV6*::RUNX1* (*n* = 19), *TCF3::PBX1* (*n* = 7), *BCR::ABL1* (*n* = 5), *KMT2A*::V (*n* = 10), *IGH*::V (*n* = 7), V::*CRLF2* (*n* = 11), iAMP21 (*n* = 8), and deletions involving *CDKN2A/B* (*n* = 38), *TP53* (*n* = 7), *RB1* (8), *ATM* (*n* = 1), and *ETV6* (*n* = 15). Hypodiploidy (*n* = 2), high‐hyperdiploidy (*n* = 38), low‐hyperdiploidy (*n* = 16), and 1q gain (*n* = 14) were also identified.

**Conclusions:**

Our findings reveal a cytogenomic landscape characterized by a predominance of high‐risk abnormalities such as iAMP21 and *KMT2A*::V, together with a lower frequency of low‐risk alterations like *ETV6::RUNX1*. The frequent coexistence of secondary abnormalities further supports the relevance of comprehensive cytogenomic profiling for accurate risk assessment. The high diagnostic coverage and rapid turnaround of the FISH‐based approach underscore its value as a reliable and efficient diagnostic tool in newly diagnosed ALL.

**Trial Registration:**

The authors have confirmed clinical trial registration is not needed for this submission

**Abbreviations**
ALL: acute lymphoblastic leukemia
EFS: event‐free survival
FISH: fluorescence in situ hybridization
HeH: high‐hyperdiploidy (51–68 chromosomes)
HeL: low‐hyperdiploidy (47–50 chromosomes)
NAD: no abnormality detected
OS: overall survival
TSG: tumor suppressor genes

## 1 Introduction

Acute lymphoblastic leukemia (ALL) is the most common pediatric malignancy and shows a particularly high incidence among Hispanic populations. While survival rates in high‐income countries exceed 90%, largely due to risk‐adapted therapies informed by clinical and cytogenomic features [1, 2, 3], outcomes in Mexico remain substantially lower (45%–85%) [4, 5]. This disparity likely reflects the combined influence of multiple structural factors, including limited diagnostic capacity, treatment interruptions, socioeconomic barriers, and constraints in supportive care. In this context, the present study aimed to characterize the incidence and spectrum of cytogenomic abnormalities in pediatric ALL patients from Western Mexico.

## 2 Materials and Methods

This study was approved by the Ethics and Scientific Committees of the Instituto Mexicano del Seguro Social (Project R‐2021‐785‐028) and included 170 pediatric patients diagnosed with ALL and treated at the Pediatric Hospital of the National Medical Center of the West–IMSS between January 2020 and April 2024. Of these, 150 (88%) had B‐Cell lineage ALL, 10 (6%) T‐Cell lineage, and 10 (6%) mixed phenotype. Bone marrow samples were collected at initial diagnosis, except in 29 patients enrolled at relapse. All patients received chemotherapy according to the Total XV protocol (St. Jude Children's Research Hospital).

Samples were processed after 2–4 h of culture in RPMI 1600 using a standard chromosome preparation protocol. Although systematic GTG‐banding was not performed, available metaphases aided in clarifying certain interphase FISH findings. The initial FISH panel of probes consisted of IGH/BCL2, TCF3/PBX1/HLF, ETV6/RUNX1, BCR/ABL1/ASS1, CRLF2, KMT2A, TP53/ATM, CDKN2A/B, RB1, and centromeric markers (Figure S1), enabling the detection of gene fusions, deletions, partial gains, and aneuploidy. Additional FISH analyses were performed to clarify ambiguous findings (Figure S1). For instance, cases showing three IGH signals with the IGH/BCL2 probe were further evaluated using an IGH break‐apart probe to distinguish between Trisomy 14 and *IGH* rearrangements. Likewise, detection of more than two BCL2 signals prompted analysis with the D18Z1 centromeric probe to differentiate between polysomy or partial gain. Samples with IGH break‐apart signal separation were subsequently tested for *IGH::CCND1*, *IGH::FGFR3*, *IGH::MAF*, *CRLF2*, or *MYC* fusions.

Suspected 1q gain, indicated by three PBX1 signals using the TCF3/PBX1/HLF probe, was confirmed with CKS1B/CDKN2C and subtelomeric 1p/1q probes. The TCF3 break‐apart probe was used to confirm suspected *TCF3* deletions, unbalanced *TCF3::PBX1* fusions, or complex rearrangements.

CRLF2 break‐apart analyses showing signal separation or multiple fused signals were further evaluated with IGH break‐apart as described in González Arreola et al. [6], or with DXZ1/DYZ1 centromeric and XpYp subtelomeric probes. Cases with proximal *CRLF2* deletion and normal X/Y centromeric counts were classified as harboring the *P2RY8::CRLF2* fusion.

Samples with KMT2A break‐apart signal separation were further analyzed using dual‐fusion probes to identify common *KMT2A* partners, including *AFF1, MLLT1, MLLT3, MLLT4*, and *MLLT10*.

Atypical ETV6::RUNX1 signal patterns were clarified by sequential FISH using the centromeric D12Z3 probe on the same nuclei or other probes (Figure S1). This allowed discrimination among Monosomy 12, partial or complete deletion of the *RUNX1::ETV6* hybrid gene, gain of an extra copy of the *ETV6::RUNX1* fusion, deletion of the non‐translocated *ETV6* allele, Trisomy 21, and intrachromosomal amplification of chromosome 21 (iAMP21). To distinguish between Polysomy 21 and iAMP21, samples were further tested with D13/21Z1, TMPRSS2/ERG/ABCG1, and subtelomeric 21q probes. In cases with suspected iAMP21, at least one metaphase cell was analyzed to confirm its appearance as a homogeneously staining region.

FISH analysis included the evaluation of at least 200 cells per sample, comprising interphase and metaphase cells when available. Cut‐off values for deletions, gains, and losses were established according to international guidelines [7], with a maximum normal threshold of 10% for *TP53* deletion. All abnormalities reported here were present in ≥ 30% of the cells analyzed. The description of cytogenomic findings was performed in accordance with the ISCN 2024 recommendations [8].

Overall survival (OS) was defined as the time from diagnosis to death or last follow‐up. Survival analyses were conducted using Kaplan–Meier curves and log‐rank tests (*p* < 0.05), with data processed in IBM SPSS Statistics v24. For this analysis, the 29 cases first enrolled at relapse were excluded.

## 3 Results

A total of 170 bone marrow samples from pediatric ALL patients were analyzed: 141 at initial diagnosis and 29 at relapse. The mean age at diagnosis was 6.6 years (±5) and the median value of 5. Forty‐nine patients were older than 10 years and 6 were younger than 1 year (Table S1). Immunophenotyping identified B‐Cell lineage in 150 cases, T‐Cell in 10, and mixed phenotype in 10. These subgroups were further stratified by cytogenomic profile (Table S1).

The initial FISH panel detected abnormalities in 143 of 170 cases (84%), including gene fusions, deletions, partial gains, and aneuploidies. No abnormalities were detected in 27 cases (16%) (NAD group). Key findings by subgroup are summarized in Table 1.

**TABLE 1:** Relevant observations in each group of cytogenomic alterations in 170 ALL patients.

**Abnormality (*n*)**	**Important remarks**	**Number of cases**
*ETV6::RUNX1* fusion (19)	As the sole abnormality (*ETV6*x2,*RUNX1*x3)(*ETV6* con *RUNX1*)x1,(D12Z3)x2^a^ (*ETV6*x2,*RUNX1*x3)(*ETV6* con *RUNX1*)x2,(D12Z3)x2^b^ (*RB1*)x1 *(CDKN2A/B)x1* +21	1 5 3 3 7 3
*TCF3::PBX1* fusion (7)	As the sole abnormality (*CDKN2C,CKS1B*)x2,(*PBX1*x3,*TCF3*x2)(*PBX1* con *TCF3*)x1^c^	4 5
*BCR::ABL1* fusion (7)	As the sole abnormality (*ASS1*x1,*ABL1*x2,*BCR*x2)(*ABL1* con *BCR*)×1^d^ (*ASS1*x2,*ABL1*x4,*BCR*x4)(*ASS1/ABL1* con *BCR*)x1(*ABL1* con *BCR*)x2^e^ *(CDKN2A/B)x1* High risk according to NCI criteria	4 3 2 3 6
*KMT2A::V* fusion (11)	As the sole abnormality Diagnosis before two years of age *KMT2A::AFF1* *KMT2A::MLLT1* *KMT2A::MLLT1* and T‐Cell ALL *KMT2A::MLLT10* t(3;11)(q2?8;q23)(*KMT2A::?*)	8 6 5 2 1 2 1
*IGH::V* fusion (5)	As the sole abnormality t(8;14)(q?1;q32)(*IGH::?CEBPD*) and DS t(14;14)(q?1;q32)(*IGH::?CEBPE*) and T‐Cell ALL *IGH::?*; two B‐Cell and one mixed	3 1 1 3
*IGH::CRLF2* fusion (7)	As the sole abnormality	4
*P2RY8::CRLF2* fusion (4)	As the sole abnormality Patient with DS (3’*CRLF2*x3,5’*CRLF2*x1)(*3’CRLF2* con *5’CRLF2)*x1,(DXZ1)x3^f^ (3’*CRLF2*x3, 5’*CRLF2*x1)(*3’CRLF2* con *5’CRLF2)*x1, (DXZ1x2, DYZ3x1)^g^	1 3 2 1
iAMP21 (8)	As the sole abnormality (subtel(XpYp))x3, (*CRLF2*)x3, (DXZ1, DYZ3)x1, (subtel(XqYq))x2^h^ Complex complement (≥3 abnormalities)^i^ *(CDKN2A/B)x1*	2 2 4 3
*CDKN2A/B* deletion (46)	As the sole abnormality: monoallelic As the sole abnormality: biallelic COA: monoallelic COA: biallelic Biallelic deletion and T‐Cell ALL (*ETV6)x1* (*RB1*)x1	4 7 21 14 6 8 9
*TP53* deletion (7)	As the sole abnormality (*ETV6)x1*	2 3
*RB1* deletion (10)	COA (*DLEU1‐2*)x1 (*CDKN2A/B*)x1 (*ETV6*)x1	10 5 9 3
*ATM* deletion (4)	COA Mixed immunophenotype	4 3
*ETV6* deletion (20)^j^	As the sole abnormality Biallelic *(CDKN2A/B)x1*	4 1 8
1q gain (15)^k^	HeH [≥51 chromosomes] HeL [47 to 50 chromosomes] (*CDKN2C*x2, *CKS1B*x3), (*PBX1*)x3, (subtel(1q))x3 (*CDKN2C*x2, *CKS1B*x2), (*PBX1*)x3, (subtel(1q))x3 (subtel(1p)x2, subtel(1q)x3) (subtel(1p)x2, subtel(1q)x2) (subtel(1p)x1, subtel(1q)x3) (subtel(1p)x2, subtel(1q)x1) Subtelomeric FISH study not done	13 2 12 1 4 6 1 2 2
HeH [51 to 68 chromosomes] (41)	Only chromosomal gains Mixed immunophenotype 1q gain *(CDKN2A/B)*x1	22 3 13 9
HeL [47 to 50 chromosomes] (18)	Only chromosomal gains Coexisting with gene fusions iAMP21 1q gain Tumor suppressor gene deletions	5 5 3 2 3
Hypodiploid complement (2)^l^	Hipodiploidy (35 chromosomes/70 chromosomes) Hiperhaploidy (25 chromosomes)	1 1
NAD (27)	High risk according to NCI criteria T‐Cell immunophenotype Mixed immunophenotype	20 1 3

*Note*: Several patients have more than one abnormality. V = variable partner gene. COA = Coexisting with other abnormalities. NAD = No abnormality detected. DS = Down syndrome. Abnormalities inferred from specific cytogenomic formulas: (a) unbalanced *ETV6::RUNX1* fusion with 3′ *ETV6* deletion; (b) *ETV6::RUNX1* fusion with deletion of the non‐translocated *ETV6* allele; (c) unbalanced *TCF3::PBX1* fusion; (d) unbalanced *BCR::ABL1* fusion with deletion of *ASS1, 5′ ABL1*, and *3′ BCR* sequences; (e) Balanced *BCR::ABL1* fusion with an extra copy of the *BCR::ABL1* gene; (f–g) gain of the X chromosome harboring the *P2RY8::CRLF2* fusion; (h) gain of pseudoautosomic region 1 sequences. (i) [9]. (j) The five cases with the formula (*ETV6x2,RUNX1x*3)(*ETV6* con *RUNX1*)x1 were not included in this counting. (k) The five cases with the formula (*CDKN2C,CKS1B*)x2,(*PBX1*x3,*TCF3*x2)(*PBX1* con *TCF3*)x1 were not included in this counting. (l) In these cases, FISH analysis was complemented with GTG‐banding. The chromosomal translocations t(3;11), t(8;14), and t(14;14) were not identified by GTG‐banding; they were revealed through consecutive FISH analysis of metaphase cells using KMT2A or IGH break‐apart probes followed by centromeric probes targeting the unknown derivative chromosome.

### 3.1 B‐Cell ALL

**FIGURE 1:**
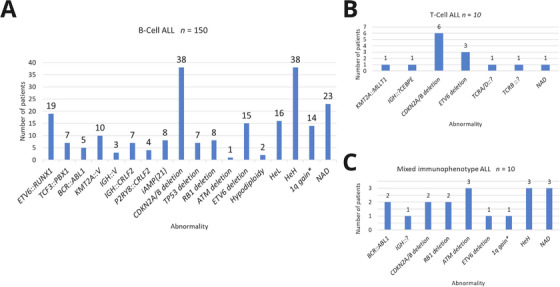
Cytogenomic findings. (A) Distribution of cytogenomic abnormalities observed in 150 patients with B‐Cell ALL. (B) Cytogenomic results obtained from 10 patients with T‐Cell ALL. (C) Cytogenomic abnormalities detected in 10 patients with ALL and mixed immunophenotype. *Five cases with 1q gain linked to the unbalanced *TCF3::PBX1* gene fusion were not included in the 1q gain counting. V (variable partner gene); HeL (low hyperdiploidy): 47–50 chromosomes; HeH (high hyperdiploidy): 51–68 chromosomes; NAD = no abnormality detected.

Seventeen abnormalities were identified among the 150 B‐Cell ALL cases, with a variable number of alterations per patient. The most frequent findings were *CDKN2A/B* deletions (38/150), HeH (38/150), and NAD (23/150) (Figure 1A). Fusions and/or iAMP21 were detected in 63 patients (42%), and 70 tumor suppressor gene (TSG) deletions were seen in 51 patients.

**FIGURE 2:**
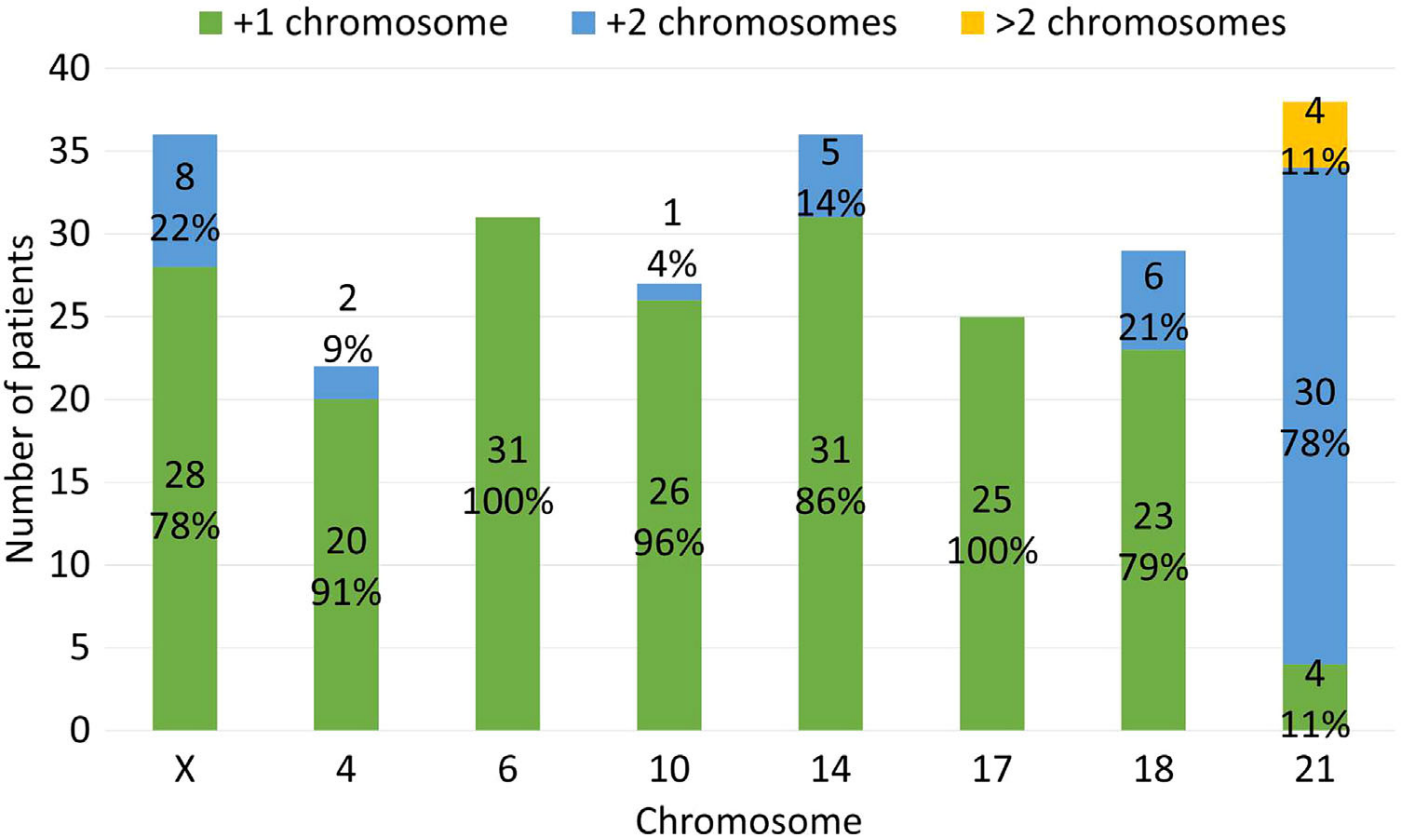
Chromosome gains observed in 38 B‐Cell ALL patients with HeH.

Among HeH cases, the most frequent gained chromosomes were 21, X, and 14, with Tetrasomy 21 being the most recurrent HeH‐associated alteration (Figure 2).

### 3.2 T‐Cell ALL

Seven cytogenomic abnormalities were detected in 10 T‐Cell ALL cases (0–3 per patient). The most frequent were *CDKN2A/B* biallelic deletions (6/10) and *ETV6* deletions (3/10) (Figure 1B).

### 3.3 Mixed Phenotype ALL

Nine abnormalities were identified in 10 patients (0–2 per case). The most frequent findings were *ATM* deletions, HeH, and NAD, each present in three patients (Figure 1C).

### 3.4 Overall Survival

After excluding the 29 cases recruited at relapse, the mean follow‐up time of the remaining patients (*n* = 141) was 19 months (range, 0–72; median, 19). The mean OS was 54.9 months (95% CI, 49.5–60.3), with an estimated 5‐year OS of 73%. Forty patients experienced relapse (29 at inclusion and 11 after diagnosis), and 45 patients died (1–95 months after diagnosis). Fourteen deaths (31%) occurred within the first month, and seven additional deaths occurred by Month 3, resulting in a total of 21 early deaths.

No significant differences in OS were observed among cytogenomic subgroups, immunophenotypic categories, age groups, sexes, white blood cell counts, or National Cancer Institute (NCI) risk classifications.

## 4 Discussion

Using an extended panel of eleven FISH assays per patient, we identified cytogenomic abnormalities in 143 of 170 cases (84%), while 27 cases (16%) showed no detectable alterations. This contrasts sharply with most ALL studies in Mexico and globally, which rely on RT‐PCR for only the four most common gene fusions (*BCR::ABL1, TCF3::PBX1, KMT2A::AFF1*, and *ETV6::RUNX1*), yielding informative results in just 19%–45% of patients [10, 11, 12, 13, 14, 15]. As a result, a substantial proportion of patients remain without defined cytogenomic risk, limiting personalized treatment decisions.

FISH offers broader detection capabilities than RT‐PCR. It can identify key genomic imbalances, including additional copies of *BCR::ABL1* and *ETV6::RUNX1* gene fusions, partial gains (e.g., 1q), unbalanced fusions (e.g., *TCF3::PBX1*), and numerical alterations such as hypo‐ or hyperdiploidy. Most of these findings are often missed by karyotyping or DNA index analysis, which depend on metaphase quantity, quality, and other technical variables [16, 17]. Notably, this approach also revealed concurrent alterations in individual patients, such as gene fusions coexisting with TSG deletions and/or aneuploidies, underscoring the complex cytogenomic landscape observed in some cases.

### 4.1 *ETV6::RUNX1* Fusion

*ETV6::RUNX1* was the most frequent fusion, detected in 19 of 150 B‐Cell ALL cases (12.6%). This rate is comparable to reports from India and Taiwan but lower than those from the UK, Korea, other European cohorts, and the USA (Table 2). These findings are consistent with previous Mexican series, confirming that this favorable‐risk marker is underrepresented in our population [10, 11, 12, 15, 18, 19, 20, 21].

**TABLE 2:** Frequencies of genetic abnormalities in B‐ALL.

Author [reference]	Population	Technique	Reported frequency	*p* value
*ETV6::RUNX1* gene fusion 19/150 (12.6%)				
Harrison et al. [22]	UK	FISH	447/2027 (22%)	< 0.0093
Moorman et al. [23]	UK	FISH	368/1451 (25%)	< 0.0008
Yu et al. [14]	Taiwan	RT‐PCR	36/220 (16%)	NS
Lee et al. [13]	Korea	RT‐PCR, FISH, and karyotype	41/190 (22%)	< 0.05
Jeha et al. [24]	USA	RNA‐sequencing	128/598 (21%)	< 0.022
Hiemenz et al. [25]	USA	FISH and NGS	20/160 (12.5%)	NS
Olsson et al. [26]	Sweden	SNP array	58/260 (22%)	0.022
Siraj et al. [27]	India	RT‐PCR	18/259 (7%)	NS
Compilation USA (2000)^a^	USA	RT‐PCR	170/773 (22%)	< 0.014
Compilation Europe (2000)^a^	Europe	RT‐PCR	422/1838 (23%)	< 0.005
Our *ETV6::RUNX1* fusion frequency was similar to those reported in other studies of the Mexican population [10, 11, 15, 18, 20, 28, 29]				
*KMT2A::AFF1* gene fusion 5/150 (3.3%)				
Harrison et al. [22]	UK	FISH	16/2016 (0.8%)	< 0.009
Moorman et al. [23]	UK	FISH	17/1627 (1%)	< 0.042
Lee et al. [13]	Korea	RT‐PCR	14/190 (7%)	NS
Yu et al. [14]	Taiwan	RT‐PCR	8/220 (4%)	NS
Siraj et al. [27]	India	RT‐PCR	0/259 (0)	< 0.0065^b^
Compilation USA (2000)^a^	USA	RT‐PCR	67/5590 (1%)	< 0.04^b^
Compilation Europe (2000)^a^	Europe	RT‐PCR	63/3926 (2%)	NS
Friedrich P et al. [28]	Mexico	FISH	21/505 (4%)	NS
Moreira DC et al. [29]	Mexico	FISH, RT‐PCR	40/934 (4%)	NS
Flores‐Lujano et al. [18]	Mexico	RT‐PCR	5/512 (1%)	NS
Mata‐Rocha et al. [11]	Mexico	RT‐PCR	7/247 (3%)	NS
Juarez‐Avendaño et al. [15]	Mexico	RT‐PCR	5/154 (3%)	NS
Bekker‐Méndez et al. [10]	Mexico	RT‐PCR	3/282 (1%)	NS
*KMT2A::V* 10/150 (6.6%)				
Yu et al. [14]	Taiwan	RT‐PCR and karyotyping	14/220 (6%)	NS
Jeha et al. [24]	USA	FISH and RNA‐seq	28/598 (5%)	NS
Hiemenz et al. [25]	USA	Karyotype, FISH, and NGS	5/160 (3%)	NS
Olsson et al. [26]	Sweden	SNP array	10/260 (4%)	NS
Moorman et al. [23]	UK	FISH	30/1627 (2%)	< 0.0005
Harrison et al. [22]	UK	FISH	47/2016 (2%)	< 0.0034
Perez‐Vera et al. [20]	Mexico	FISH and karyotype	5/57 (9%)	NS
*IGH::V* gene fusions 10/150 (6.6%)				
Hiemenz et al. [25]	USA	FISH	3/160 (2%)	< 0.047^b^
Russell et al. [30]	UK	FISH	94/2546 (4%)	NS
iAMP21 8/150 (5.3%)				
Hiemenz et al. [25]	USA	Karyotype and FISH	7/160 (4%)	NS
Jeha et al. [24]	USA	FISH	5/598 (0.83%)	< 0.001
Virk et al. [31]	India	FISH	5/478 (1%)	< 0.0039
Lee et al. [13]	Korea	FISH	2/190 (1%)	< 0.05
Yu et al. [14]	Taiwan	MLPA	4/214 (2%)	NS
Olsson et al. [26]	Sweden	SNP array	2/260 (0.77%)	< 0.012
Moorman et al. [23]	UK	FISH	29/1449 (2%)	< 0.022
Friedrich P et al. [28]	Mexico	FISH	43/505 (8.5%)	NS
Moreira DC et al. [29]	Mexico	FISH	14/220 (6.4%)	NS
*IGH::CRLF2* and *P2RY8::CRLF2* 11/150 (7.3%)				
Hiemenz et al. [25]	USA	Expression studies and FISH	31/160 (19%)	< 0.0034
Jeha et al. [24]	USA	FISH and RNA‐seq	22/598 (4%)	NS
Juárez‐Velázquez et al. [12]	Mexico	Expression studies and FISH	51/133 (38%)	< 0.0001

Abbreviation: *V*, variable partner gene.

^a^ Data published in *Leukemia* (2000; 14: 2196–2320) and cited from the compilation presented in tab. 2 of Siraj et al. [27]. Comparisons were made with Yates corrected chi‐square test.

^b^ Fisher exact test.

The well‐documented association between *ETV6::RUNX1* and favorable prognosis [3, 23, 32] suggests that its reduced frequency may partially account for the less favorable survival outcomes reported in Mexican pediatric ALL cohorts. Within the *ETV6::RUNX1* subset, additional alterations were common: deletions involving the 3′*ETV6* sequence occurred in 5 of 19 cases, *CDKN2A/B* deletions in 7, and *RB1* loss, deletion of the non‐translocated *ETV6* allele, and Chromosome 21 gain in 3 cases each (Table 1). These co‐occurring lesions may have biological relevance and merit further investigation.

### 4.2 *TCF3::PBX1* Fusion

The *TCF3::PBX1* fusion was identified in 7 of 150 B‐Cell ALL cases (4.6%), representing a significantly higher frequency than the 1.6% (74/4516) reported in a European cohort (*p* < 0.005) (cited from tab. 2 of Siraj et al. [27]), but not differing from the 5% (384/7640) reported in a Chinese population [33]. Notably, five of these cases corresponded to the unbalanced form of the rearrangement (Table 1). The prognostic significance of balanced versus unbalanced *TCF3::PBX1* fusions remains controversial. While several studies have not consistently demonstrated significant differences in outcome between the two forms [33], Chang et al. [34] reported fewer relapses among patients harboring the unbalanced rearrangement. Further studies are warranted to elucidate the biological and clinical implications of this structural variant.

### 4.3 *BCR::ABL1* Fusion

The *BCR::ABL1* fusion, a well‐recognized high‐risk marker in ALL [3], was found in 7 of 170 patients: five with B‐Cell (3.3%) and two with mixed‐lineage immunophenotypes (Table S1). Four cases showed the fusion as the sole abnormality, whereas three also carried *CDKN2A/B* deletions and an additional *BCR::ABL1* copy (Table 1). This frequency (3.3%) was consistent with reports from the USA, Asia, and previous Mexican studies [10, 11, 12, 13, 14, 15, 27]. In addition, deletions involving the *ASS1*–5′*ABL1*–3′*BCR* region were observed in three cases.

### 4.4 KMT2A

*KMT2A* rearrangements were identified in 11 of 170 patients: 10 with B‐Cell and one with T‐Cell immunophenotype. The detected fusions included *KMT2A::AFF1* (5 cases), *KMT2A::MLLT1* (3), *KMT2A::MLLT10* (2), and one with an unidentified partner (*KMT2A*::?) related to a t(3;11)(q?28;q23) translocation (Figure 1A,B and Table 1). These high‐risk rearrangements are typically associated with early age at onset and elevated leukocyte counts [24]. In our cohort, 6 of the 11 patients were diagnosed before 2 years of age, and 8 presented leukocyte counts > 50,000/µL (Table S1). The overall frequency observed in B‐Cell ALL cases (10/150; 6.6%) exceeded that reported in the UK [22, 23], but was comparable to data from Taiwan, Sweden, the USA, and Mexico City. Notably, the *KMT2A::AFF1* fusion accounted for 3.3% of B‐Cell cases, a rate higher than those from the UK, India, and the USA, but consistent with other Mexican series (Table 2).

### 4.5 iAMP21

iAMP21 was detected in 8 of 150 B‐Cell ALL patients (5.3%) (Figure 1A). The common amplified region spanned ∼3.7 Mb from *RUNX1* to *ERG*, consistent with previous copy number profiling studies [22, 35]. In two cases, iAMP21 appeared as the sole abnormality; the other six exhibited complex cytogenomic profiles (≥ 3 abnormalities), as described in prior reports [9, 36, 37]. Associated alterations included +X, –4, +11, +13, –13, and deletions in *ETV6* and *CDKN2A/B* (Table 1). Although *P2RY8::CRLF2* fusions were not detected, gain of the pseudoautosomal region 1 (PAR1) was observed in two cases, suggesting *CRLF2* overexpression [21, 25, 31].

The observed iAMP21 frequency (5.3%) was significantly higher than that reported in the USA, UK, India, Sweden, and Korea, but aligned with rates in Taiwan and Mexico (Table 2).

### 4.6 IGH and CRLF2 Rearrangements

The *IGH::CRLF2* and *P2RY8::CRLF2* fusions are associated with Ph‐like B‐Cell ALL and poor prognosis [12, 25, 30]. *IGH::CRLF2* was detected in seven patients, four as the sole abnormality. *P2RY8::CRLF2* was found in four cases, three of them with Down syndrome (Table 1), consistent with prior reports [38]. In addition, three patients (two with Down syndrome) showed a gain of the deleted X chromosome carrying the fusion. This rare secondary event has only been documented three times in the Mitelman Database [39], all reported by Olson et al. [26]. Other *IGH* rearrangements involving alternative partners were identified in five additional patients (Table 1). The overall *IGH* rearrangement frequency in our cohort was 6.6% (Figure 1A), not significantly different from that reported in a UK series (4%), but it was from the USA cohort (2%) (Table 2) [30].

### 4.7 TSG Deletions

Unlike most global cytogenomic studies in ALL, which do not routinely assess large‐scale deletions, our approach systematically screened TSGs including *CDKN2A/B, TP53, RB1, ATM*, and *ETV6* [40, 41, 42]. Among B‐Cell ALL patients, relapse or death occurred in 30 of 55 with at least one TSG deletion, compared to 30 of 80 without deletions (*p* = 0.074). Kaplan–Meier analysis revealed no significant difference in survival.

*TP53* deletions were identified in seven patients, two of whom had no other abnormalities. *ATM* deletions were found in four patients, three with mixed immunophenotypes. Although both alterations have been linked to poor prognosis [43, 44], only two of the 40 relapsed cases harbored *TP53* deletions, and none had *ATM* deletions.

*RB1* deletions were detected in ten patients, nine of whom also had *CDKN2A/B* deletions. In five cases, co‐deletion of *DLEU1/2* was observed, indicating loss of a ∼2.5 Mb region that includes *miR‐15a* and *miR‐16‐1*, alterations linked to poor prognosis in chronic lymphocytic leukemia [45, 46].

*ETV6* deletions were found in 20 patients, four as the sole abnormality and co‐occurring with *ETV6::RUNX1* in three patients and with *CDKN2A/B* deletions in eight. Its prognostic significance remains uncertain.

*CDKN2A/B* deletion was the most frequent abnormality, detected in 46/170 cases (38/150 B‐Cell ALL; Figure 1A), consistent with previous Mexican reports [20, 47]. In T‐Cell ALL, it was observed exclusively in its biallelic form in 6/10 cases, aligning with prior studies [48], but significantly higher than reported by Friedrich et al. [28] (9/42; *p* < 0.05) (Table 2). Common co‐occurrences of *CDKN2A/B* deletion were with *RB1* (9 cases), *ETV6* (8), *ETV6::RUNX1* (7), *IGH*::V (5), and HeH (9) (Table 2). Although *CDKN2A/B* deletions have been associated with poor prognosis features, including older age, higher leukocyte counts, hepatosplenomegaly, and *BCR::ABL1* rearrangement [47, 48, 49, 50, 51], our cohort did not show such a correlation. However, the combined *RB1* and *CDKN2A/B* loss in nine cases highlights their role in tumorigenesis may through disruption of G1/S cell cycle control, contributing to poor clinical outcomes in adolescents and adults [50, 51].

### 4.8 HeH (High hyperdiploidy 51–68 Chromosomes)

We observed HeH in 41 of 170 patients (24%). Although HeH is considered a low‐risk abnormality in the WHO classification of hematolymphoid neoplasms and is typically associated with favorable clinical outcomes [16], 12 of 41 patients in our cohort experienced relapse, and 9 of 41 harbored concurrent *CDKN2A/B* deletions.

The frequency of HeH in our B‐Cell ALL cohort (38/150) did not significantly differ from those reported in the USA (154/598) [24], Korea (41/190) [13], Sweden (75/260) [26], or the UK (562/1486) [23]. In contrast, two recent studies from Mexico in Alliance with St. Jude group reported lower HeH frequencies by karyotyping 82/817 [29] and 86/505 [28], both significantly below our rate (*p* < 0.00001 and *p* < 0.031, respectively). Interestingly, in Friedrich et al. [28], applying a DNA index > 1.16 increased detection to 185/505 cases (*p* < 0.014 vs. our series), and results differed markedly between methods (*p* < 0.00001). These discrepancies highlight the influence of detection methodology on HeH prevalence, which is clinically relevant since HeH status directly affects risk classification and therapy. Moreover, given its biological heterogeneity, defining the specific chromosomal gains is essential to understand HeH‐driven leukemogenesis and its potential impact on drug response and clonal resistance.

We also identified the coexistence of 1q gain and HeH (Table 1). This structural abnormality, reported in up to 15% of HeH cases [16, 52, 53], appears to be largely confined to hyperdiploid ALL. Among the 15 patients with 1q gain, 13 co‐occurred with HeH, and 2 with low‐hyperdiploidy. In 14 of these, the breakpoint was proximal to the *CKS1B* gene, with only one case involving the region between *CKS1B* and *PBX1* (Table 1). Recently, El Ashry et al. identified 1q gain as an independent adverse prognostic factor for disease‐free survival in HeH patients [53].

### 4.9 HeL (Low Hyperdiploidy, 47–50 Chromosomes)

HeL was detected in 18 cases: five exhibited exclusively numerical chromosomal gains, while the remaining cases also harbored structural abnormalities (Table 1). This subtype constitutes approximately 10%–15% of diagnosed cases, with structural aberrations observed in nearly 75%, thereby hindering consistent risk stratification [54].

### 4.10 Hypodiploid Complements (< 46 Chromosomes)

A hypodiploid complement was identified in two patients: one hyperhaploid (24 chromosomes) and another with a doubled clone (35/70 chromosomes) (Table 1). Hypodiploidy accounts for ∼1% of pediatric ALL cases and is strongly associated with a poor prognosis [3], consistent with the early deaths observed in both patients (at 5 and 23 months, respectively).

### 4.11 NAD Group

No cytogenomic abnormalities were detected in 27 of 170 patients (16%), yet 8 of them relapsed, and 10 died (Table S1). These findings suggest the presence of additional lesions not detectable by our FISH panel. Further investigation is warranted to identify potential alterations in other genes.

### 4.12 Overall Survival

The estimated 5‐year OS in our cohort was 73%, substantially lower than international rates, which range from 85% to 95% [3, 24]. However, the statistical power of this analysis is limited by the small cohort size and the relatively short follow‐up time (mean = 19 months; range, 0–72). Comprehensive outcome analyses of individual and complex cytogenomic abnormalities will require a larger cohort and longer follow‐up periods.

## 5 Conclusions

This cohort exhibited a lower frequency of low‐risk cytogenomic alterations (such as *ETV6::RUNX1*) and a higher prevalence of high‐risk abnormalities (iAMP21, *KMT2A*::V). Notably, many cases harboring WHO‐recognized recurrent genetic alterations also presented additional tumor suppressor gene (TSG) deletions and/or secondary abnormalities, resulting in complex cytogenomic profiles with the potential to further worsen prognosis — for example, *CDKN2A/B* deletions were frequently detected even in patients carrying otherwise low‐risk markers.

The main advantages of our FISH‐based approach include its comprehensive coverage and rapid turnaround time. All recruited cases were successfully analyzed, and microscopic evaluation of the initial FISH panel was completed within 48–72 h after sample receipt, allowing for a timely and integrated cytogenomic interpretation.

An abnormal cytogenomic profile was identified in 84% of cases, whereas the remaining 16% were classified as negative for all alterations detectable with our panel. Therefore, a definitive cytogenomic diagnosis, either abnormal or negative, was achieved in all analyzed cases, underscoring the high diagnostic coverage and analytical robustness of our FISH‐based strategy.

## Funding

This work was funded by Instituto Mexicano del Seguro Social (R‐2021‐785‐028). The funding body was not involved in activities related to this manuscript.

## Ethics Statement

This study was approved by the Ethics and Scientific Committees of the Instituto Mexicano del Seguro Social (Project # R‐2021‐785‐028).

## Consent

All participants’ parents were informed of the study objectives and invited to participate on a voluntary basis. Bone marrow collection was performed concurrently with routine procedures for initial morphological, immunological, and cytogenetic diagnosis. Verbal informed consent was obtained from all parents, and the protocol received approval from the Institutional Ethics Committee (Project # R‑2021‑785‑028).

## Conflicts of Interest

The authors declare no conflicts of interest.

## Supporting information




**Supporting Table 1**: jha270220‐sup‐0001‐tableS1.docx.

**Supporting Figure 1**: jha270220‐sup‐0002‐figureS1.docx.

## Data Availability

Data are available from the corresponding author upon reasonable request and institutional ethics committee approval.
