# Supplementary material for: Cytogenomic Abnormalities in Children With Acute Lymphoblastic Leukemia From Western Mexico: A Single‐Center Fluorescence In Situ Hybridization‐Based Study
Source: EJHaem. 2026 Jan 19;7(1):e70220. doi: 10.1002/jha2.70220 (PMC12814622; doi:10.1002/jha2.70220)
Supplement: Supplementary file 1 — Supporting Table 1: jha270220‐sup‐0001‐tableS1.docx. [file JHA2-7-e70220-s002.docx]

Supplemental Table S1. Clinical features of patients with acute lymphoblastic leukemia (ALL) by cytogenomic subgroup.


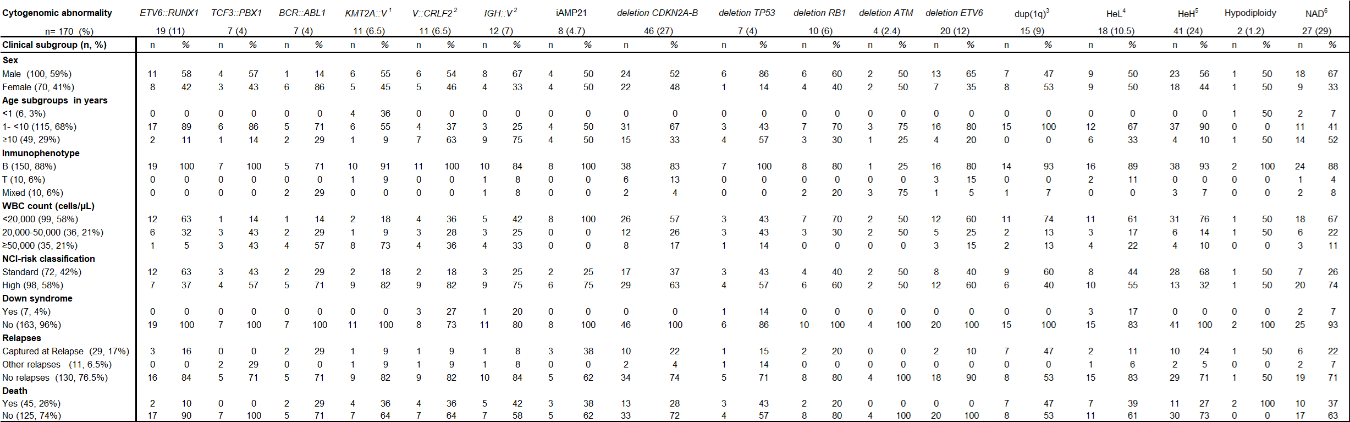


Both V*::CRLF2* and *IGH::*V columns include 7 patients with *IGH::CRLF2* gene fusion. V, variable partner gene; WBC, white blood cell; NCI, National Cancer Institute.
