# Supplementary material for: Cytogenomic Abnormalities in Children With Acute Lymphoblastic Leukemia From Western Mexico: A Single‐Center Fluorescence In Situ Hybridization‐Based Study
Source: EJHaem. 2026 Jan 19;7(1):e70220. doi: 10.1002/jha2.70220 (PMC12814622; doi:10.1002/jha2.70220)

Supplementary Figure S1. Information related to FISH probes included in the initial panel as well as the probes used in the confirmatory studies.


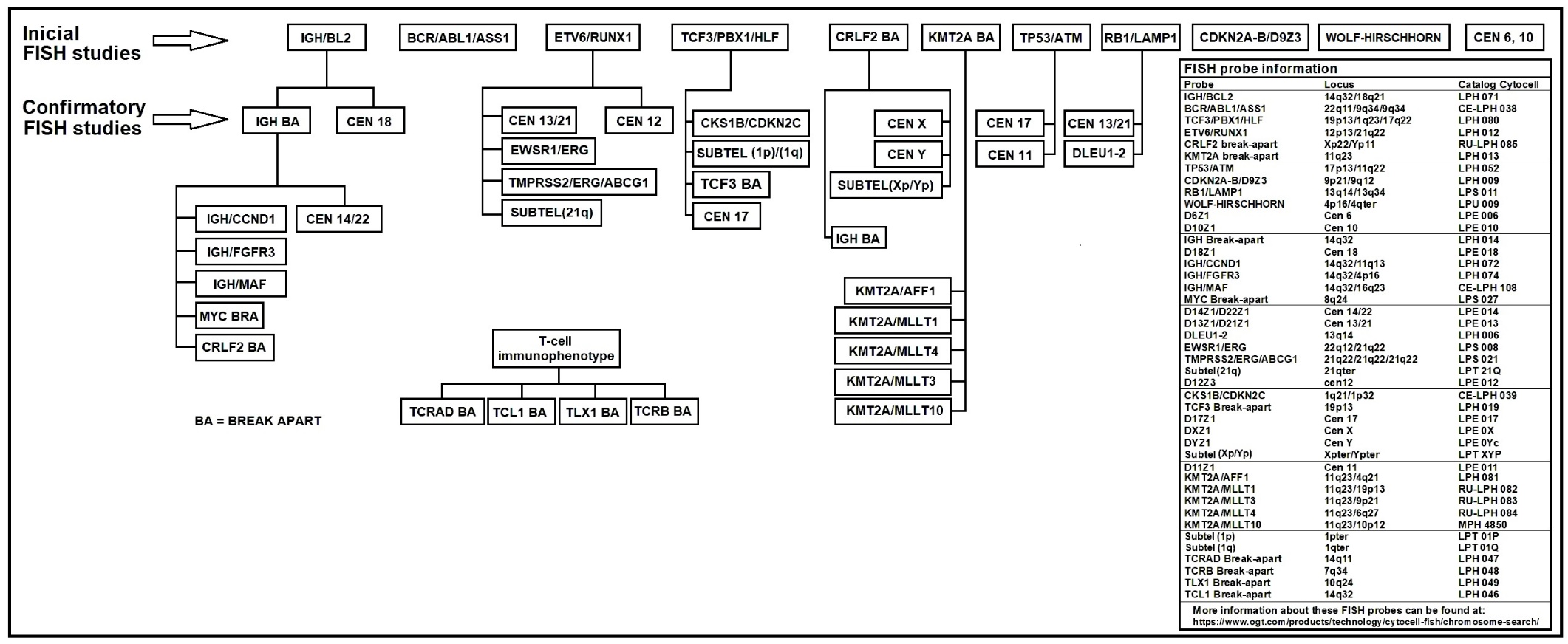

Supplement: Supplementary file 2 — Supporting Figure 1: jha270220‐sup‐0002‐figureS1.docx. [file JHA2-7-e70220-s001.docx]
